# Maternal serum uric acid concentration and pregnancy outcomes in women with pre‐eclampsia/eclampsia

**DOI:** 10.1002/ijgo.12697

**Published:** 2018-11-08

**Authors:** Tam M. Le, Long H. Nguyen, Nam L. Phan, Duong D. Le, Huy V.Q. Nguyen, Vinh Q. Truong, Thanh N. Cao

**Affiliations:** ^1^ Department of Obstetrics and Gynecology Hue University of Medicine and Pharmacy Hue University Hue Vietnam; ^2^ Department of Public Health Hue University of Medicine and Pharmacy Hue University Hue Vietnam

**Keywords:** Eclampsia, Intrauterine growth restriction, Neonatal complications, Pre‐eclampsia, Preterm birth, Uric acid

## Abstract

**Objectives:**

To determine the relationship between maternal serum uric acid levels and fetal/neonatal complications in women with pre‐eclampsia/eclampsia, and to establish a predictive threshold value.

**Methods:**

A diagnostic test and historical cohort study conducted by prospective cross‐sectional data collection on pregnant women with pre‐eclampsia/eclampsia at Hue University Hospital, Vietnam, between March 2015 and July 2017. Pre‐eclampsia was diagnosed based on ACOG criteria. Serum uric acid levels were measured by enzymatic colorimetric testing using a Cobas c 501 analyzer (Roche Diagnostics, Mannheim, Germany). Fetal complications included intrauterine growth restriction, preterm delivery, fetal death, and neonatal death.

**Results:**

There were 205 women enrolled. Serum uric acid at a cutoff of 393 μmol/L is a good predictor of fetal/neonatal complications (AUC 0.752), with 64.4% sensitivity and 79.5% specificity. High uric acid level (≥393 μmol/L) resulted in increased risk of preterm birth (OR 6.367, 95% CI 3.009–13.084), low Apgar scores (OR 5.514, 95% CI 1.877–16.198), intrauterine growth restriction (OR 7.188, 95% CI 3.592–14.382), and neonatal death (OR 7.818, 95% CI 1.614–37.867). There was no relationship between uric acid level and fetal death (OR 1.803, 95% CI 0.355–9.168).

**Conclusions:**

Maternal serum uric acid concentration is a good predictor of fetal/neonatal outcomes in women with pre‐eclampsia/eclampsia.

## Abstract 2

Maternal serum uric acid concentration is a good predictor of fetal/neonatal outcomes in pregnant women with pre‐eclampsia/eclampsia.

## 1 INTRODUCTION

Uric acid is the final substance in the process of purine metabolism.^1^, ^2^ Elevated levels are considered to be an early biomarker of kidney damage in women with pre‐eclampsia and also a factor in predicting fetal death.^1^, ^3^ The role of uric acid as the possible cause of maternal and fetal deaths in pre‐eclamptic patients (10%–15% and 5.9%, respectively) is controversial and the subject of ongoing study.^3^

In addition to complications for the mother, pre‐eclampsia can also result in serious consequences for the fetus, including fetal distress, intrauterine growth restriction (IUGR), and preterm or perinatal death. Although most studies have shown that maternal uric acid levels play an important role in the prognosis of pre‐eclampsia,^4^, ^5^, ^6^ a unified threshold value has not been determined. Threshold values of 6 mg/dL (530.4 μmol/L) and 5.6 mg/dL for 38 weeks of gestation (521.4 μmol/L) have been reported^5^, ^7^; while Parrish et al.^8^ reported a mean uric acid level of 363.4 μmol/L in pregnancies with adverse outcomes.

In Vietnam, although there have been many studies on pre‐eclampsia in terms of prevention, early diagnosis, and pregnancy outcomes, there are limited studies that have investigated uric acid concentration and its role in predicting severe fetal complications in pre‐eclamptic patients. The aim of the present study was to determine the relationship between maternal serum uric acid levels and fetal/neonatal complications in women with pre‐eclampsia/eclampsia, and to establish a predictive value for these complications.

## 2 MATERIALS AND METHODS

A diagnostic test and historical cohort study conducted by prospective cross‐sectional data collection on singleton pregnant women with pre‐eclampsia/eclampsia admitted to the Department of Obstetrics and Gynecology, Hue University Hospital, Vietnam, between March 2015 and July 2017. The study excluded chronic hypertensive women, patients with metabolic disorders, and those with diseases that adversely affect serum uric acid levels in pregnancy, such as chronic kidney disease, acute and chronic nephritis, nephrotic syndrome, and kidney failure. Patients with cellular damage as a result of cancer and hemolytic diseases were also excluded. The study was approved by the Hue University of Medicine and Pharmacy Ethics Committee. Because uric acid sampling is indicated routinely for pregnant women with pre‐eclampsia in our clinical practice, informed consent was not required to obtain samples.

To estimate the sample size required for this study, we used the equation n=*Z*_α/2_^2^ × *P* × (1 − *P*)/Δ2, where α=0.05, Δ=0.05, and *Z*_α/2_=1.96. Based on the complication rate of intrauterine fetal death, neonatal death, small for gestational age or prematurity from the study by Livingston et al.^9^ the expected prevalence taken from small for gestational age, which was the most common complication, was 46/125 (*P*=11%). The minimum sample size required to identify the fetal/neonatal outcomes for women with pre‐eclampsia attending our hospital was 150 patients.

Recruited patients were divided into two groups: pre‐eclampsia (group 1) or severe pre‐eclampsia and eclampsia (group 2), based on the criteria from the American College of Obstetricians and Gynecologists (ACOG).^10^ Pre‐eclampsia was defined as the onset of hypertension and proteinuria from 20 weeks of pregnancy onward.^10^ Severe pre‐eclampsia was defined as pre‐eclampsia and at least one of the following symptoms: systolic blood pressure greater than or equal to 160 mm Hg or diastolic blood pressure greater than or equal to 110 mm Hg, pulmonary edema, cerebral or visual disorder, thrombocytopenia, liver dysfunction, epigastric or right upper quadrant pain, nonresponse to treatment, serum creatinine concentration greater than 1.1 mg/dL, or double serum creatinine concentration. Eclampsia was defined as the occurrence of seizures with stages according to diagnostic criteria.^10^

Fetal complications included IUGR, preterm delivery, fetal death, and neonatal death. Neonatal death was defined as death occurring during the first 4 weeks after birth, while fetal death was defined as intrauterine death before delivery (stillbirth).^11^

Each patient was measured for height and weight. Body mass index (BMI) was calculated as body weight in kilograms divided by the square of height in meters. Serum uric acid levels were drawn intravenously at the hospital after each patient had rested for 30 minutes. The sterilization principle and compliance preservation regime were ensured during testing by use of the comparison color method (enzymatic colorimetric test) using a Coba c 501 analyzer (Roche Diagnostics, Mannheim, Germany) with UA2 ACN 700 (serum/plasma). The peroxide reacts in the presence of peroxidase, *N*‐ethyl‐*N*‐(2‐hydroxy‐3‐sulfopropyl)‐3‐methylaniline, and 4‐aminophenazone to form a quinonediimine dye. The intensity of the red color formed is proportional to the uric acid concentration and is determined photometrically; the methods used followed the manufacturer's instructions.

All analyses were performed using SPSS version 2.0 (IBM, Armonk, NY, USA). Receiver operating characteristic (ROC) curves were constructed to examine the diagnostic test performance of uric acid for the prognosis of each fetal/neonatal outcome, and general complications defined as at least one of five events occurring, including: preterm birth, APGAR score less than 7 in the first minute, fetal death, IUGR, or neonatal death. Groups were compared using the *t* test for independent samples. Values are given as mean and standard deviation (SD) or absolute number and percentage. Results are expressed as odds ratio (OR) with 95% confidence interval (CI) or two‐sided *P* value. *P*<0.05 was considered statistically significant.

## 3 RESULTS

During the study period, 205 pregnant women with pre‐eclampsia/eclampsia met the eligibility criteria and were recruited consecutively. The general characteristics of the study population and the fetal/neonatal complications are shown in Table 1. The mean age of the study participants was 30.6 ± 6.7 years, and there was no significant difference in age between group 1 (pre‐eclampsia) and group 2 (severe pre‐eclampsia/eclampsia). Differences in parity and BMI were also not significant between the groups.

**Table 1:** General characteristics of the study population and fetal/neonatal outcomes.^a^

Characteristics	Group 1: Pre‐eclampsia (n=112)	Group 2: Severe pre‐eclampsia/eclampsia (n=93)	Total (n=205)	*P* value
Maternal age, y	30.45 ± 6.99	30.88 ± 6.27	30.6 ± 6.7	0.675
<35	78 (69.6)	63 (67.7)	141 (68.8)	0.888
≥35	34 (30.4)	30 (32.3)	64 (31.2)	
Parity				0.421
Nulliparous	54 (48.2)	42 (45.2)	105 (51.2)	
Multiparous	58 (51.8)	51 (54.8)	100 (48.8)	
BMI	27.68 ± 3.95	26.41 ± 3.45	27.23 ± 3.82	0.577
Uric acid, μmol/L	328.99 ± 88.49	427.60 ± 113.54	364.11 ± 108.72	<0.001
Preterm birth	7 (6.3)	39 (41.9)	46 (22.4)	<0.001
Apgar score <7	0	18 (20.7)^b^	18 (9.0)^b^	<0.001
Fetal death	0	6 (6.5)	6 (2.9)	0.008
IUGR	15 (13.4)	38 (40.9)	53 (25.9)	<0.001
Neonatal death	0	10 (10.8)	10 (4.9)	<0.001

Abbreviations: BMI, body mass index (calculated as weight in kilograms divided by the square of height in meters); IUGR, intrauterine growth restriction.

a Values are given as mean ± SD or number (percentage) unless otherwise indicated.

b Fetal deaths were not included for the purpose of calculating percentages.

Uric acid concentration was significantly higher in group 2 compared with group 1 (427.60 ± 113.54 vs 328.99 ± 88.49; *P*<0.001). IUGR was the most common fetal/neonatal complication, accounting for 25.9% (n=53), followed by preterm birth at 22.4% (n=46). Apgar score of less than 7 was established for 199 live neonates owing to 6 cases of fetal death.

**Figure 1:**
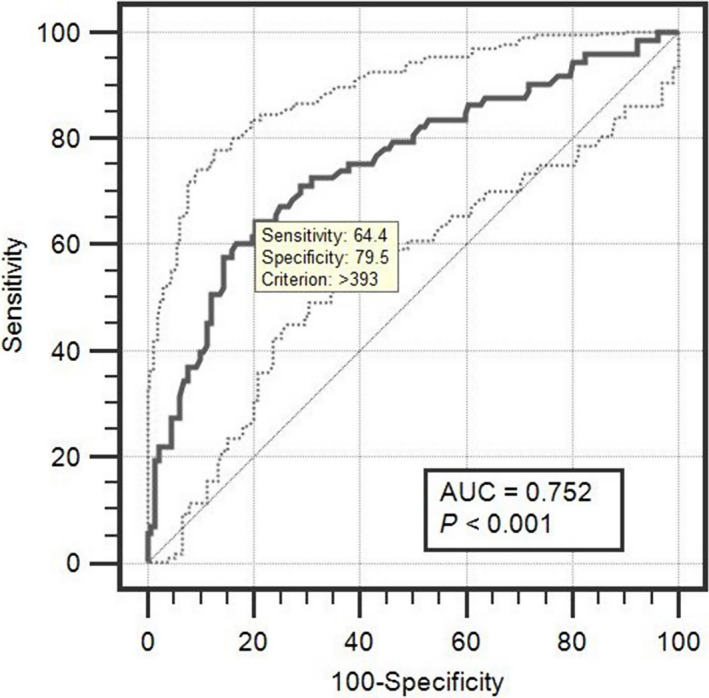
Receiver operating characteristic curve of serum uric acid concentration in cases of fetal/neonatal complications among women with pre‐eclampsia/eclampsia.

For individual fetal/neonatal complications, the AUC of uric acid was highest for prognosis of neonatal death (AUC 0.759, *P*<0.004), followed by preterm birth (AUC 0.753, *P*<0.001), Apgar score <7 (AUC 0.752, *P*<0.001), and IUGR (AUC 0.723, *P*<0.001) (Table 2). For general fetal/neonatal complications (occurrence of at least one of the other five events), the AUC was 0.752 and the threshold uric acid level was 393 μmol/L, with 64.4% sensitivity and 79.5% specificity (Fig. 1).

**Table 2:** Criterion values, sensitivity, and specificity of serum uric acid concentration as markers for prognosis of fetal/neonatal complication

Complications	Criterion of acid uric, μmol/L	Sensitivity	Specificity	AUC	*P* value
Preterm birth	>410	65.2	78.6	0.753	<0.001
Apgar <7 (n=199)	>376	83.3	61.3	0.752	<0.001
Fetal death	>376	83.3	57.3	0.634	0.169
IUGR	>393	69.8	75.7	0.723	<0.001
Neonatal death	>378	90.0	60.5	0.759	<0.004
General fetal/neonatal complications (occurrence of at least one of other five complications)	>393	64.4	79.5	0.752	<0.001

Abbreviations: AUC, area under the curve; IUGR, intrauterine growth restriction.

The relationship between fetal outcomes and uric acid threshold of 393 μmol/L is shown in Table 3. High uric acid level (≥393 μmol/L) resulted in increased risk of preterm birth (OR 6.367, 95% CI 3.009–13.084; *P*<0.001); low Apgar score (OR 5.514, 95% CI 1.877–16.198; *P*=0.002); IUGR (OR 7.188, 95% CI 3.592–14.382; *P*<0.001); and neonatal death (OR 7.818, 95% CI 1.614–37.867; *P*=0.009). The only complication for which the difference was not significant was fetal death (*P*=0.773).

**Table 3:** Relationship between fetal outcomes and uric acid threshold of 393 μmol/L

Outcomes	Uric acid ≥393 μmol/L (n=74)	Uric acid <393 (n=131)	*P* value	OR (95% CI)
Preterm birth	32 (43.2)	14 (10.7)	<0.001	6.367 (3.009–13.084)
Apgar <7 (n=199)	13 (18.3)^a^	5 (3.8)	0.002	5.514 (1.877–16.198)
Fetal death	3 (4.1)	3 (2.3)	0.773	1.803 (0.355–9.168)
IUGR	37 (50.0)	16 (12.2)	<0.001	7.188 (3.592–14.382)
Neonatal death	8 (10.8)	2 (1.5)	0.009	7.818 (1.614–37.867)

Abbreviations: CI, confidence interval; IUGR, intrauterine growth restriction; OR, odds ratio.

a Fetal deaths were not included for the purpose of calculating this percentage.

There was no association between uric acid level and maternal factors such as age, parity, or delivery method (Table 4). The only associated factor was severe pre‐eclampsia/eclampsia (OR 5.188, 95% CI 2.790–9.649; *P*<0.001).

**Table 4:** Relationship between uric acid levels and maternal factors

Maternal factors	Uric acid ≥393 μmol/L	Acid uric <393 μmol/L	*P* value	OR (95% CI)
Maternal age, y			0.615	0.811 (0.435–1.512)
≥35 (n=64)	43 (67.2)	21 (32.8)		
<35 (n=141)	88 (62.4)	53 (37.6)		
Parity			0.642	0.807 (0.473–1.482)
Multiparous (n=100)	66 (66.0)	34 (34.0)		
Nulliparous (n=105)	65 (61.9)	40 (38.1)		
Pre‐eclampsia/eclampsia grade			<0.001	5.188 (2.790–9.649)
Severe pre‐eclampsia/eclampsia (n=93)	52 (55.9)	41 (44.1)		
Mild (n=112)	22 (19.6)	90 (80.4)		
Delivery method			0.280	1.602 (0.766–3.350)
Cesarean delivery (n=162)	100 (61.7)	62 (38.3)		
Vaginal delivery (n=43)	31 (72.1)	12 (27.9)		
Total (n=205)	131 (63.9)	74 (36.1)		

Abbreviations: CI, confidence interval; OR, odds ratio.

Table 5 shows multivariable logistic regression results for predicted fetal/neonatal outcomes based on a uric acid threshold of 393 μmol/L. Once adjusted for maternal age, grade of pre‐eclampsia and parity, uric acid at a threshold of 393 μmol/L was a good prognostic marker for IUGR (OR 5.510, 95% CI 2.611–11.628); preterm birth (OR 3.910, 95% CI 1.696–9.011); neonatal death (OR 3.249, 95% CI 0.475–22.231); low Apgar score (OR 1.716, 95% CI 0.474–6.220); and for general fetal/neonatal complications (OR 4.399, 95% CI 2.137–9.052).

**Table 5:** Multivariable logistic regression for predicted fetal/neonatal outcomes based on uric acid threshold of 393 μmol/L

Outcomes	Crude OR (95% CI)	Adjusted OR (95% CI)^a^
Preterm birth	6.367 (3.009–13.084)	3.910 (1.696–9.011)
Lower Apgar score <7	5.514 (1.877–16.198)	1.716 (0.474–6.220)
Fetal death	1.803 (0.355–9.168)	0.456 (0.058–3.593)
IUGR	7.188 (3.592–14.382)	5.510 (2.611–11.628)
Neonatal death	7.818 (1.614–37.867)	3.249 (0.475–22.231)
General complications (yes/no)	7.030 (3.710–13.319)	4.399 (2.137–9.052)

Abbreviations: CI, confidence interval; IUGR, intrauterine growth restriction; OR, odds ratio.

a Serum uric acid was adjusted for maternal age, grade of pre‐eclampsia, and parity.

## 4 DISCUSSION

This cross‐sectional study of 205 pregnant women with pre‐eclampsia/eclampsia aimed to assess the role of maternal serum uric acid levels in predicting pregnancy outcomes. The study was also designed to establish a predictive threshold value of maternal uric acid levels and occurrence of fetal complications. The association of biomarkers, including uric acid, with adverse outcomes in pre‐eclamptic pregnant women is discussed in ACOG guidelines^10^; however, its utility as a diagnostic marker is still debated.

The occurrence of pregnancy outcomes in the present study were: preterm birth rate (22.4%), IUGR (25.9%), Apgar score less than 7 (9.0%), fetal death (2.9%), and neonatal death (4.9%). Other studies have reported similarly, with preterm birth ranging from 15% to 67%, IUGR ranging from 10% to 25%, and fetal mortality ranging from 1% to 2%.^2^

Concerning individual fetal/neonatal complications, the AUC of uric acid was highest for the prognosis of neonatal death, followed by preterm birth, Apgar score less than 7, and IUGR. ROC analysis showed that a serum uric acid threshold of 393 μmol/L was a good predictor of general fetal/neonatal complications (AUC 0.752), with 64.4% sensitivity and 79.5% specificity, in women with pre‐eclampsia/eclampsia.

The results of our study are similar to those of Livingston et al.,^9^ who included 1505 pre‐eclamptic pregnant women and reported that the Z‐index of uric acid was associated with adverse perinatal outcomes (OR 1.5, 95% CI 1.4–1.7); their AUC was 0.72 (95% CI 0.69–0.74).^9^ Our threshold value of 393 μmol/L is slightly higher than normal uric acid levels in the third trimester of pregnancy (184–375 μmol/L).^9^ Similarly, Tejal and Astha^5^ selected a uric acid level of 360 μmol/L to classify risk factors for maternal and fetal complications. The cutoff threshold in the present study was also higher than that of Bellomo et al.,^12^ who reported a predictive threshold for small‐for‐gestational‐age infants of 309 μmol/L (AUC 0.784, 83.7% sensitivity, 71.7%).

Based on our threshold of 393 μmol/L, the risk of preterm birth was increased 6.367 times (95% CI 3.009–13.084), which was similar to Tejal's study (OR 6.0, 95% Cl, 1.24–28.98),^5^ but higher than results reported by Hawkins et al. (OR 3.8, 95% CI 2.6–5.6).^7^ Uric acid level was also a good predictive factor for Apgar score less than 7 in the first minute (OR 5.514, 95% CI 1.877–16.198). Similarly, another study of 103 pre‐eclamptic women found that maternal serum uric acid concentration was associated with Apgar score less than 7 (OR 11.9, 95% CI 4.4–31.8).^13^ In our study, the risk of IUGR was increased 7.188 times in women with high uric acid levels (95% CI 3.592–14.382). These results were higher than those of Hawkins (OR 1.8, 95% CI 1.2–2.7)^7^ and Tejal and Astha (OR 4.04, 95% CI 1.21–13.43).^5^

In the present study, a threshold of 393 μmol/lL was not a predictive factor for fetal death (OR 1.803, 95% CI 0.355–9.168). However, high uric acid concentration increased the risk of neonatal mortality by over sevenfold (OR 7.818, 95% CI 1.614–37.867). This result is controversial owing to the wide confidence interval. In another study, maternal serum uric acid levels were increased in 86.4% of cases of perinatal.^6^ Our results are similar to Tejal and Astha (OR 20.0, 95% CI 1.13–360.29)^5^ and Yassaee^13^ who reported that high serum uric acid concentrations increased the risk of perinatal mortality by 30.4 times (OR 30.4, 95% CI 1.7–529) compared with normal uric acid levels.

The uric acid threshold in the present study was a good predictor of severity of pre‐eclampsia/eclampsia (OR 5.188, 95% CI 2.790–9.649). These findings were much higher than those of Hawkins et al.,^7^ who reported that hyperuricemia indicated an increased risk of severe hypertension (OR 1.4, 95% CI 1.0–1.9). However, there was no association between our threshold value and cesarean or vaginal delivery.

In multivariable logistic regression analysis adjusted for maternal age, grade of pre‐eclampsia, and parity, serum uric acid was a good prognostic marker at the threshold of 393 μmol/L for general fetal/neonatal complications (OR 4.399, 95% CI 2.137–9.052). For each individual outcome, serum uric acid was a good prognostic marker for IUGR, preterm birth, neonatal death, and low Apgar score.

The present study had some limitations, such as missing maternal data for preconception and prenatal care, which made the assessment of maternal risks more difficult.

In conclusion, maternal serum uric acid concentration was a good prognostic factor for monitoring and prognosis of fetal/neonatal outcomes in women with pre‐eclampsia/eclampsia. There was a relationship between high uric acid level and the risk of preterm birth, low Apgar index, IUGR, and neonatal death, but not fetal death.

## AUTHOR CONTRIBUTIONS

LMT contributed to study design, data analysis, manuscript drafting, and critical discussion. NHL, PLN, and NVQH contributed to data collection, analysis, manuscript drafting, and critical discussion. LDD contributed to data analysis, manuscript drafting, and critical discussion. TQV and CNT contributed to data interpretation, manuscript drafting, and critical discussion. All authors were involved in drafting the work or revising it critically.

## CONFLICTS OF INTEREST

The authors have no conflicts of interest.
